# Supplementary figures and images for: A generative model for evaluating missing data methods in large epidemiological cohorts
Source: BMC Med Res Methodol. 2025 Feb 8;25:34. doi: 10.1186/s12874-025-02487-4 (PMC11806830; doi:10.1186/s12874-025-02487-4)

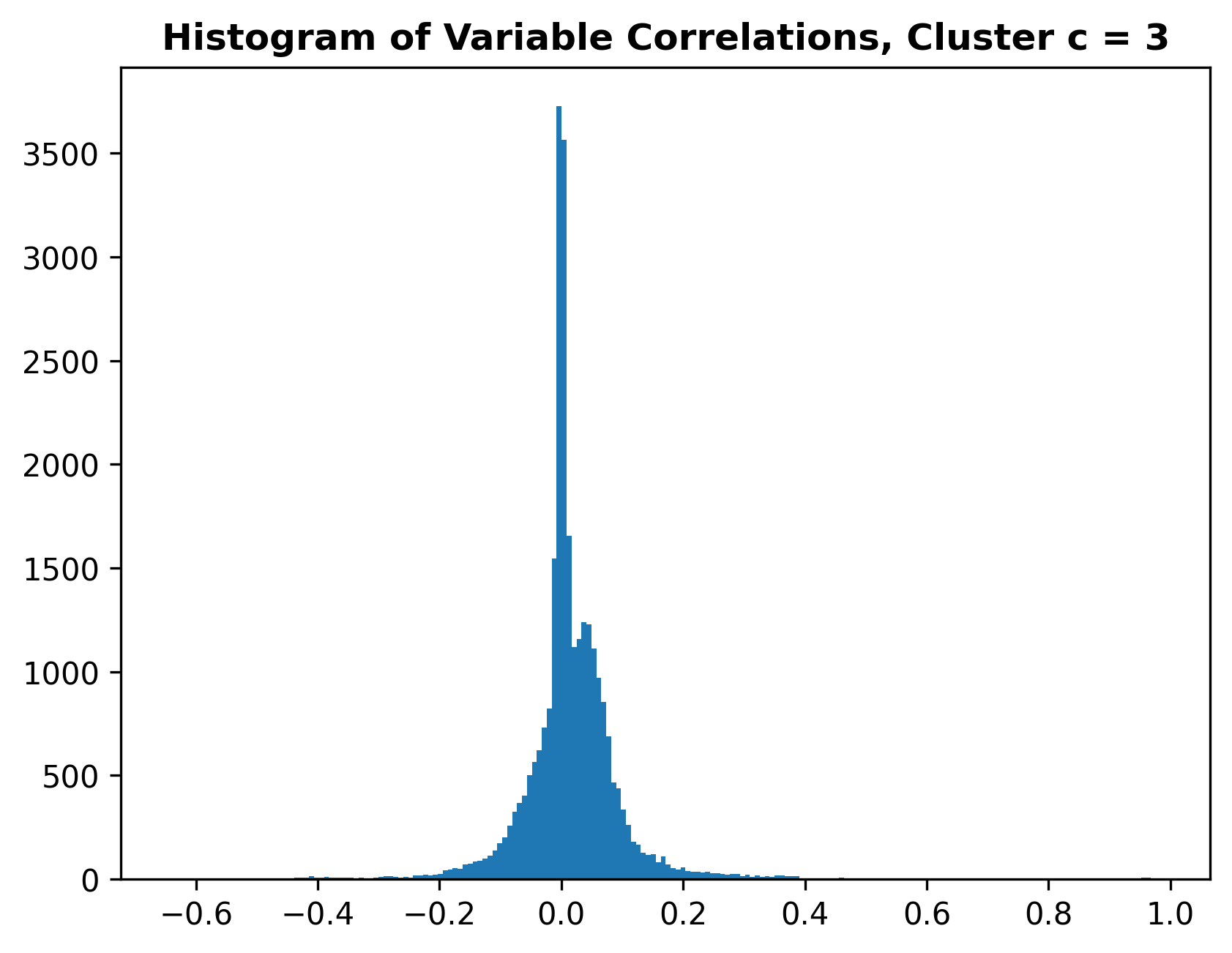

Supplement: Supplementary file 1 — Supplementary Material 1. [file 12874_2025_2487_MOESM1_ESM.zip › hist-corr-clus3.png]

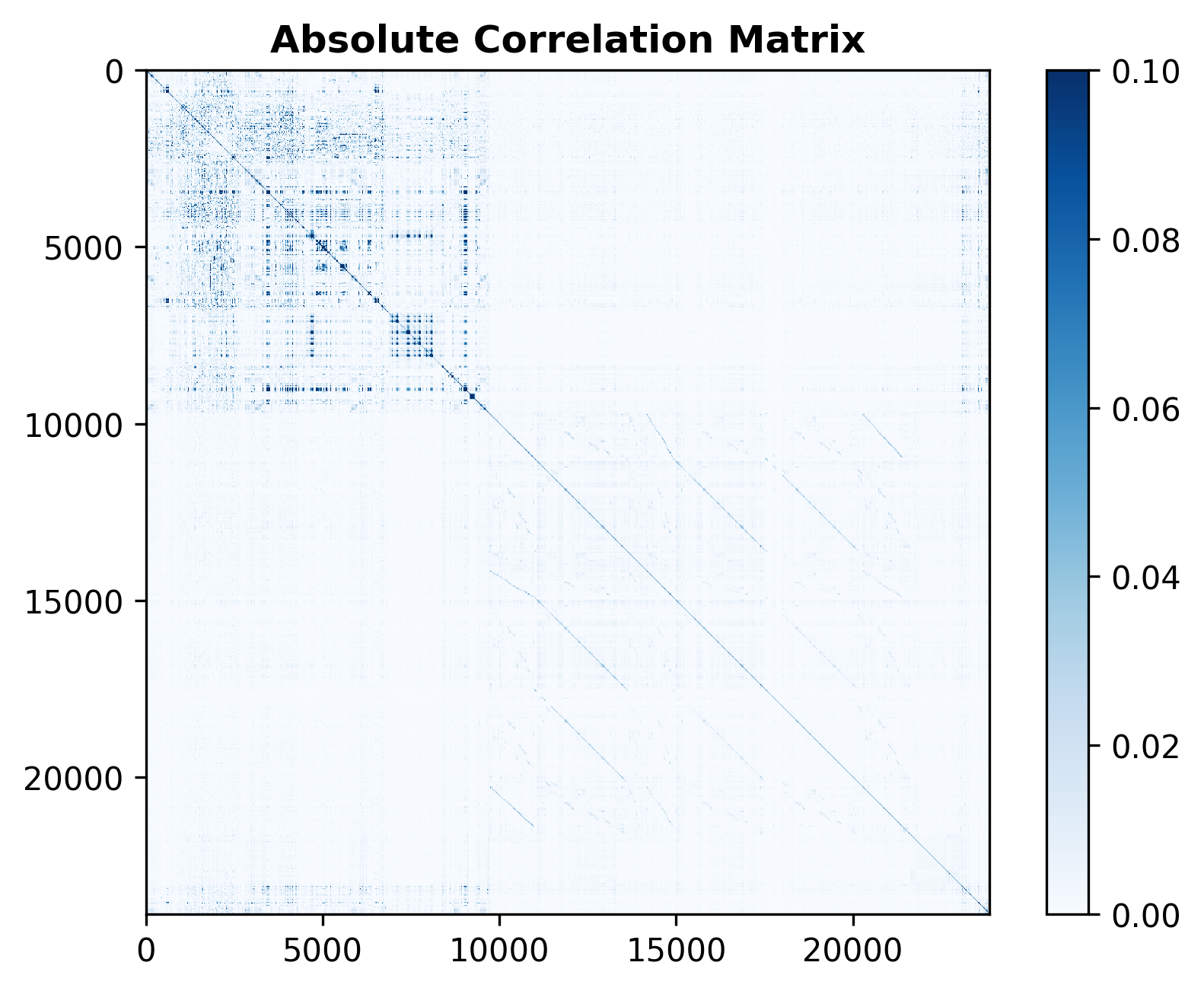

Supplement: Supplementary file 1 — Supplementary Material 1. [file 12874_2025_2487_MOESM1_ESM.zip › corr-matrix.png]

**Variable-Wise Missingness Distance Matrix**

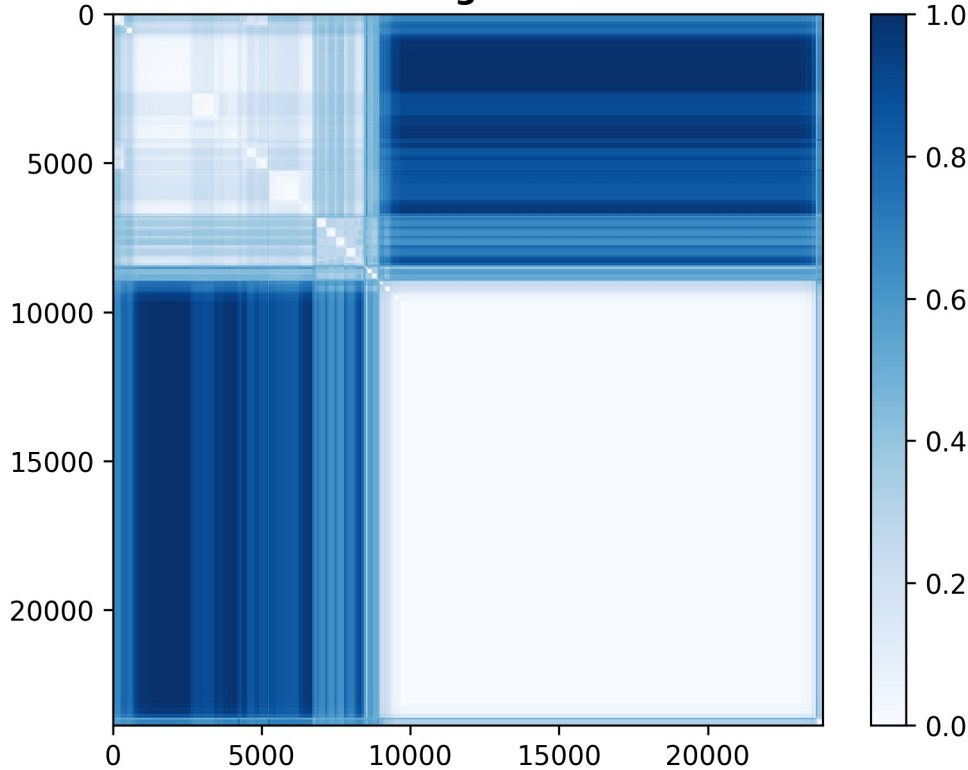

Supplement: Supplementary file 1 — Supplementary Material 1. [file 12874_2025_2487_MOESM1_ESM.zip › miss-based-matrix.pdf]

**Variable-Wise Missingness Distance Matrix, Cluster  $c = 0$**

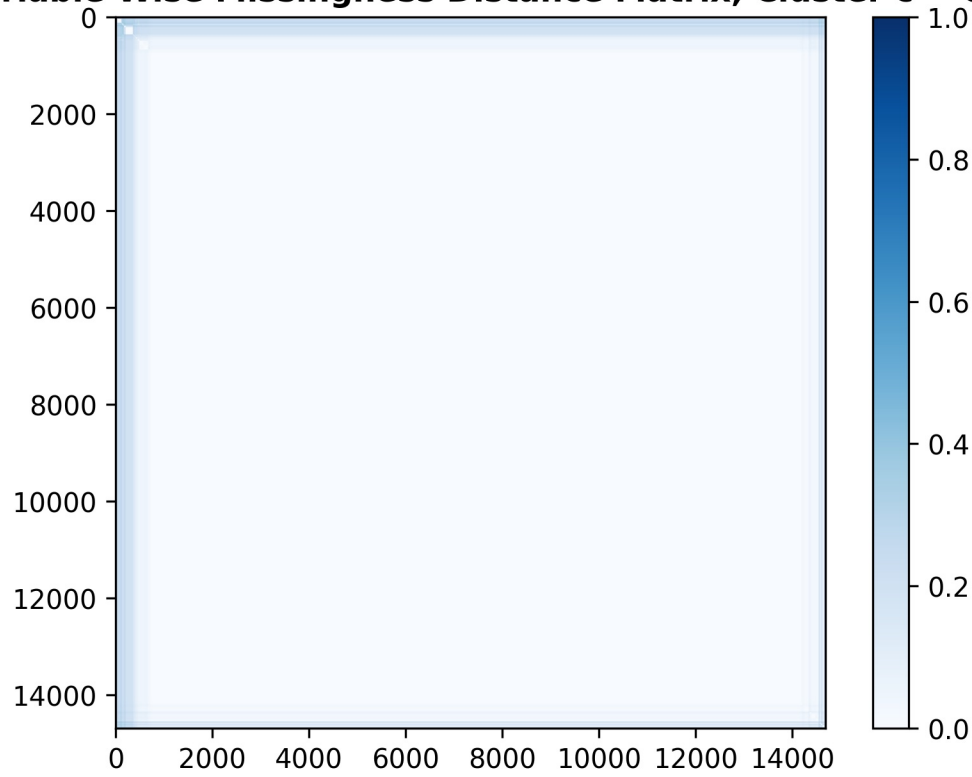

Supplement: Supplementary file 1 — Supplementary Material 1. [file 12874_2025_2487_MOESM1_ESM.zip › miss-matrix-clus0.pdf]

**Variable-Wise Missingness Distance Matrix, Cluster  $c = 1$**

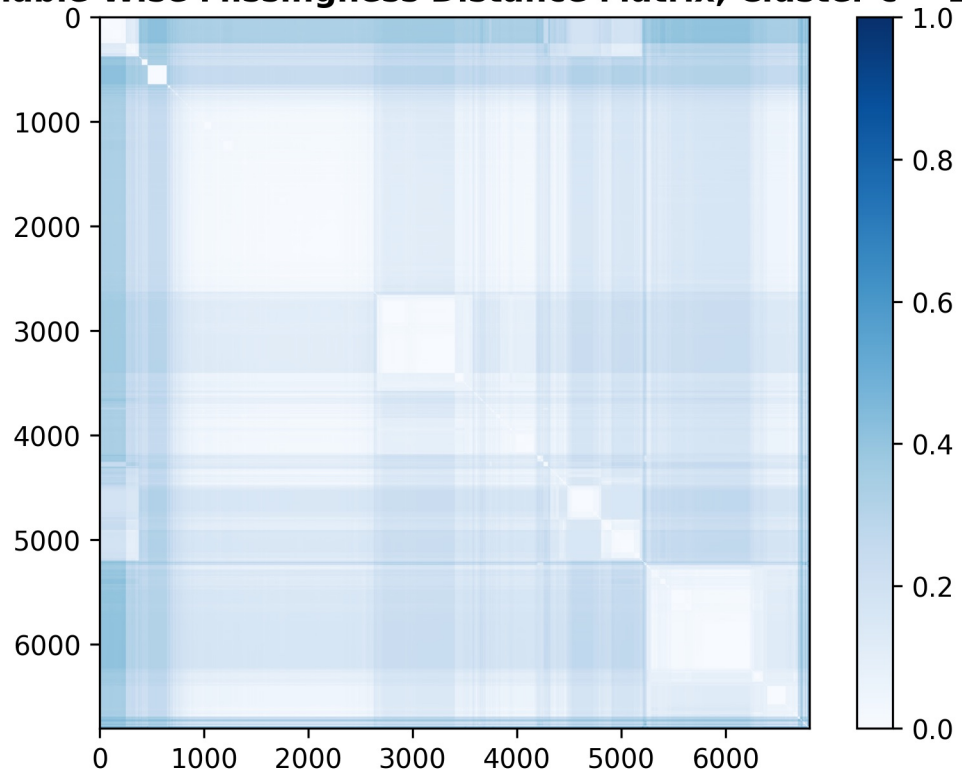

Supplement: Supplementary file 1 — Supplementary Material 1. [file 12874_2025_2487_MOESM1_ESM.zip › miss-matrix-clus1.pdf]

**Variable-Wise Missingness Distance Matrix, Cluster  $c = 2$**

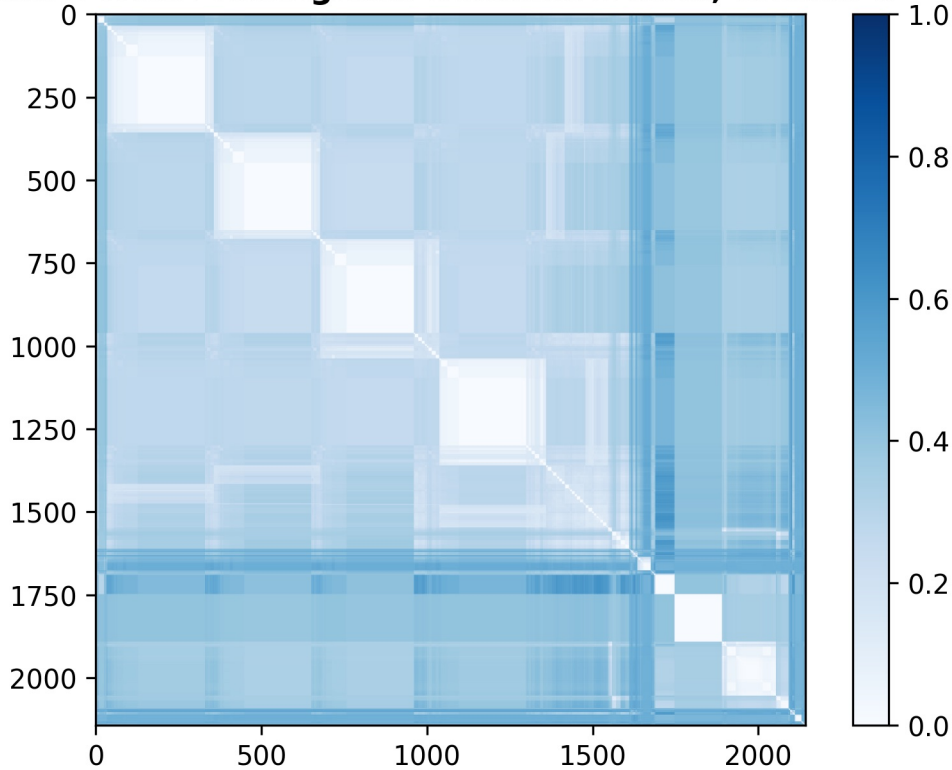

Supplement: Supplementary file 1 — Supplementary Material 1. [file 12874_2025_2487_MOESM1_ESM.zip › miss-matrix-clus2.pdf]

**Variable-Wise Missingness Distance Matrix, Cluster  $c = 3$**

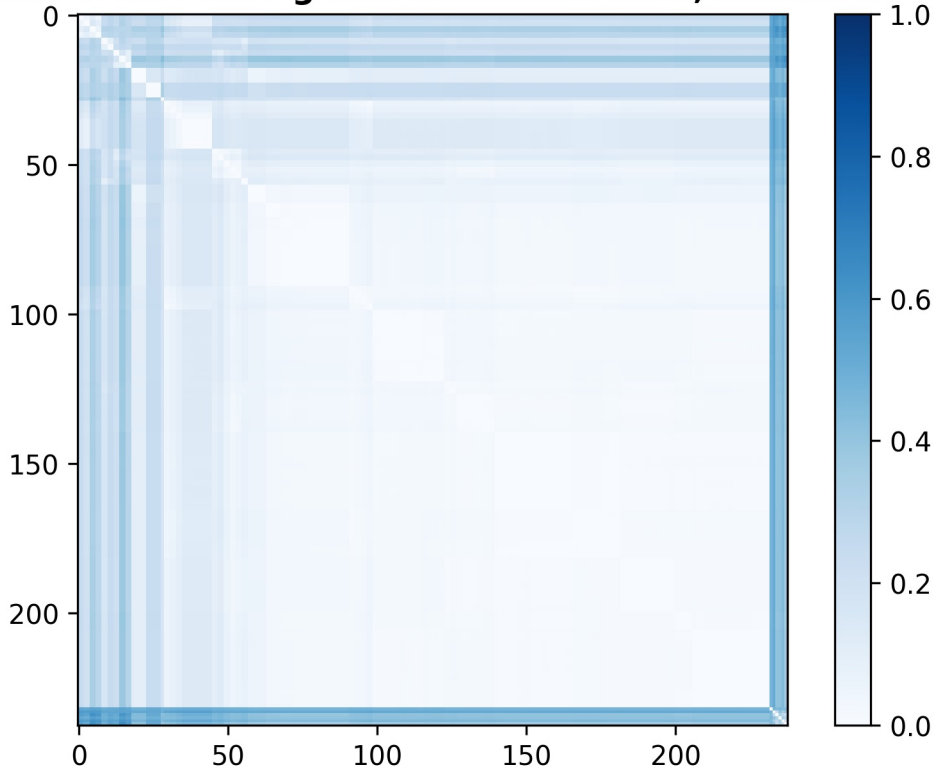

Supplement: Supplementary file 1 — Supplementary Material 1. [file 12874_2025_2487_MOESM1_ESM.zip › miss-matrix-clus3.pdf]

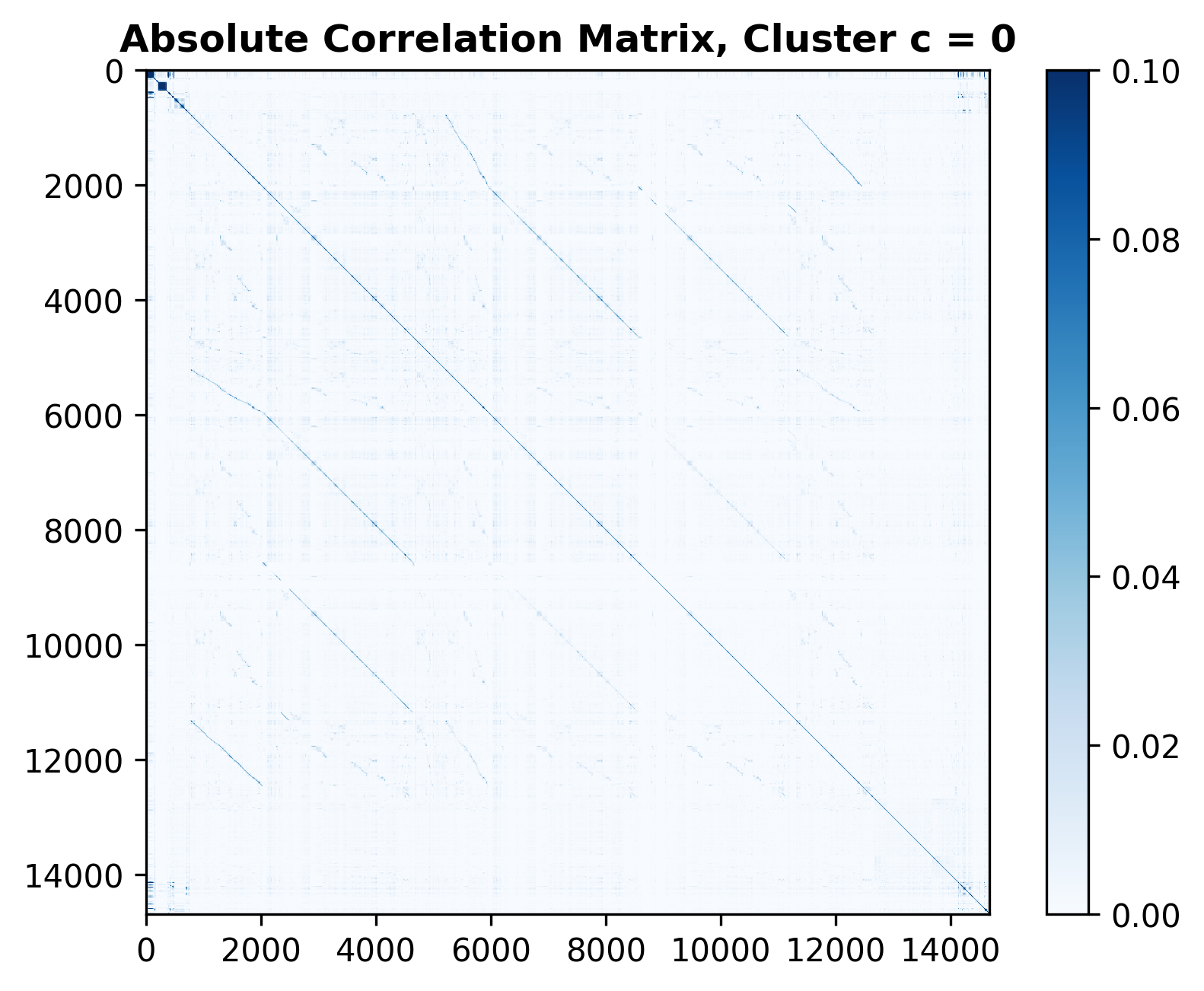

Supplement: Supplementary file 1 — Supplementary Material 1. [file 12874_2025_2487_MOESM1_ESM.zip › corr-matrix-clus0.png]

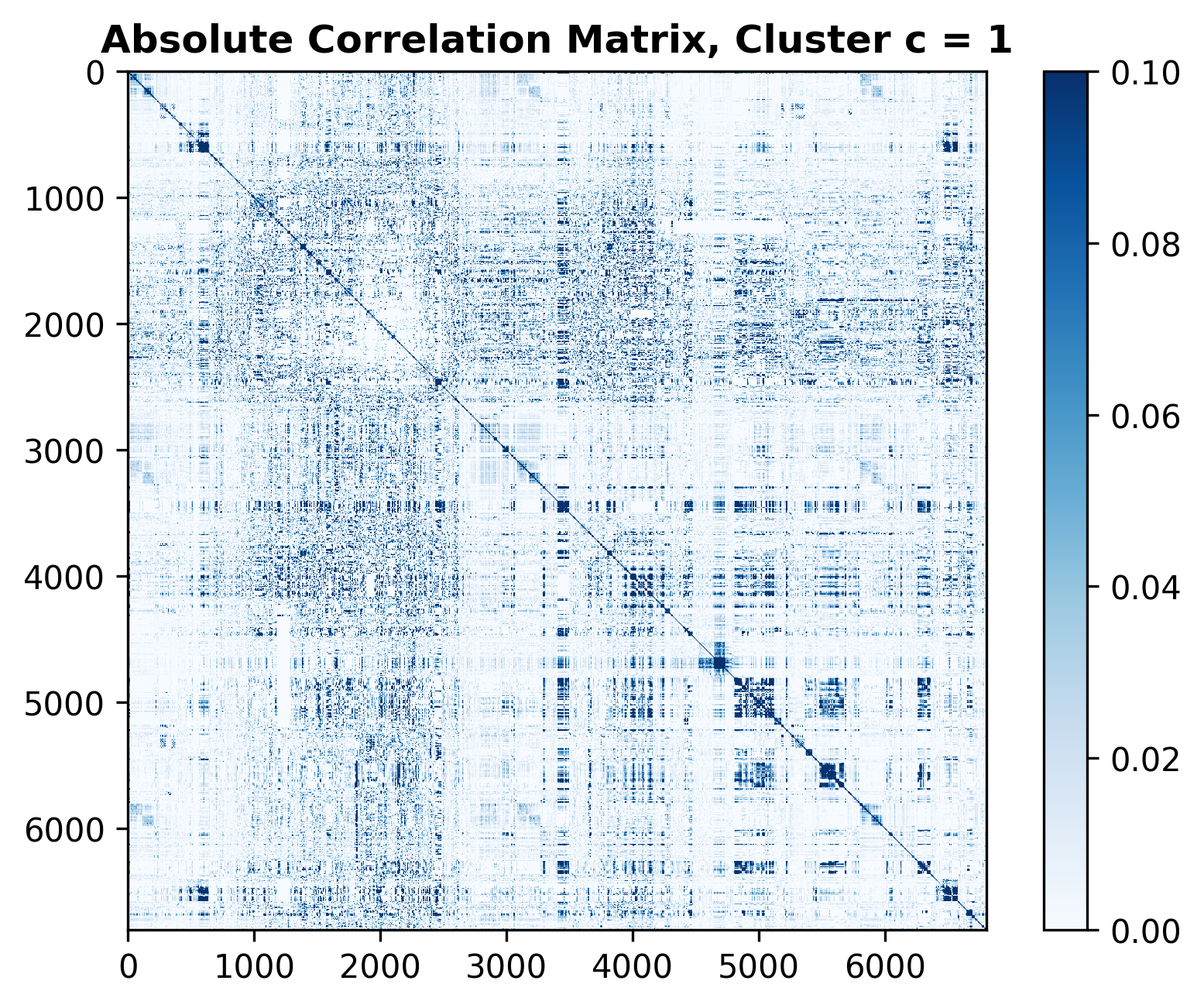

Supplement: Supplementary file 1 — Supplementary Material 1. [file 12874_2025_2487_MOESM1_ESM.zip › corr-matrix-clus1.png]

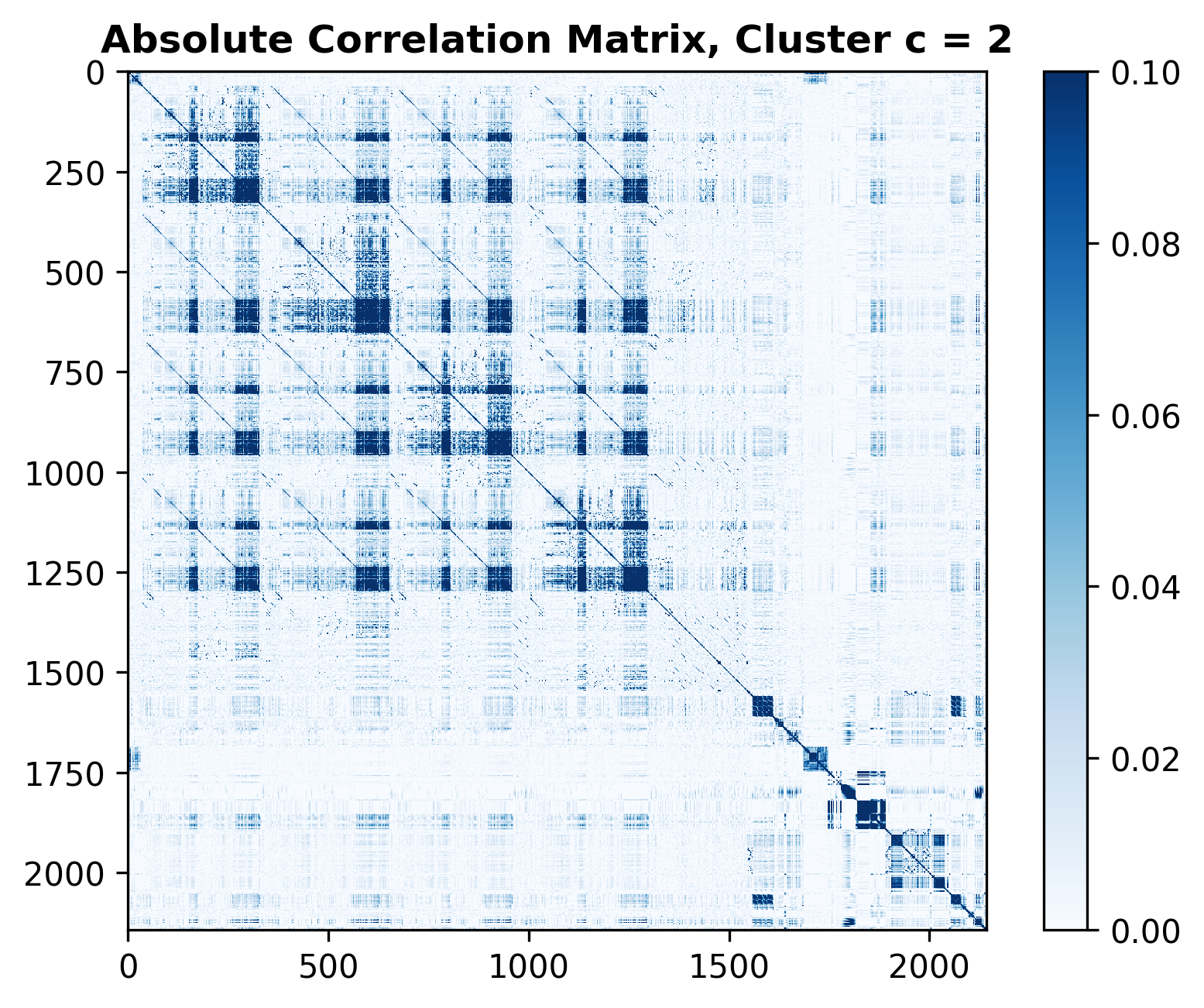

Supplement: Supplementary file 1 — Supplementary Material 1. [file 12874_2025_2487_MOESM1_ESM.zip › corr-matrix-clus2.png]

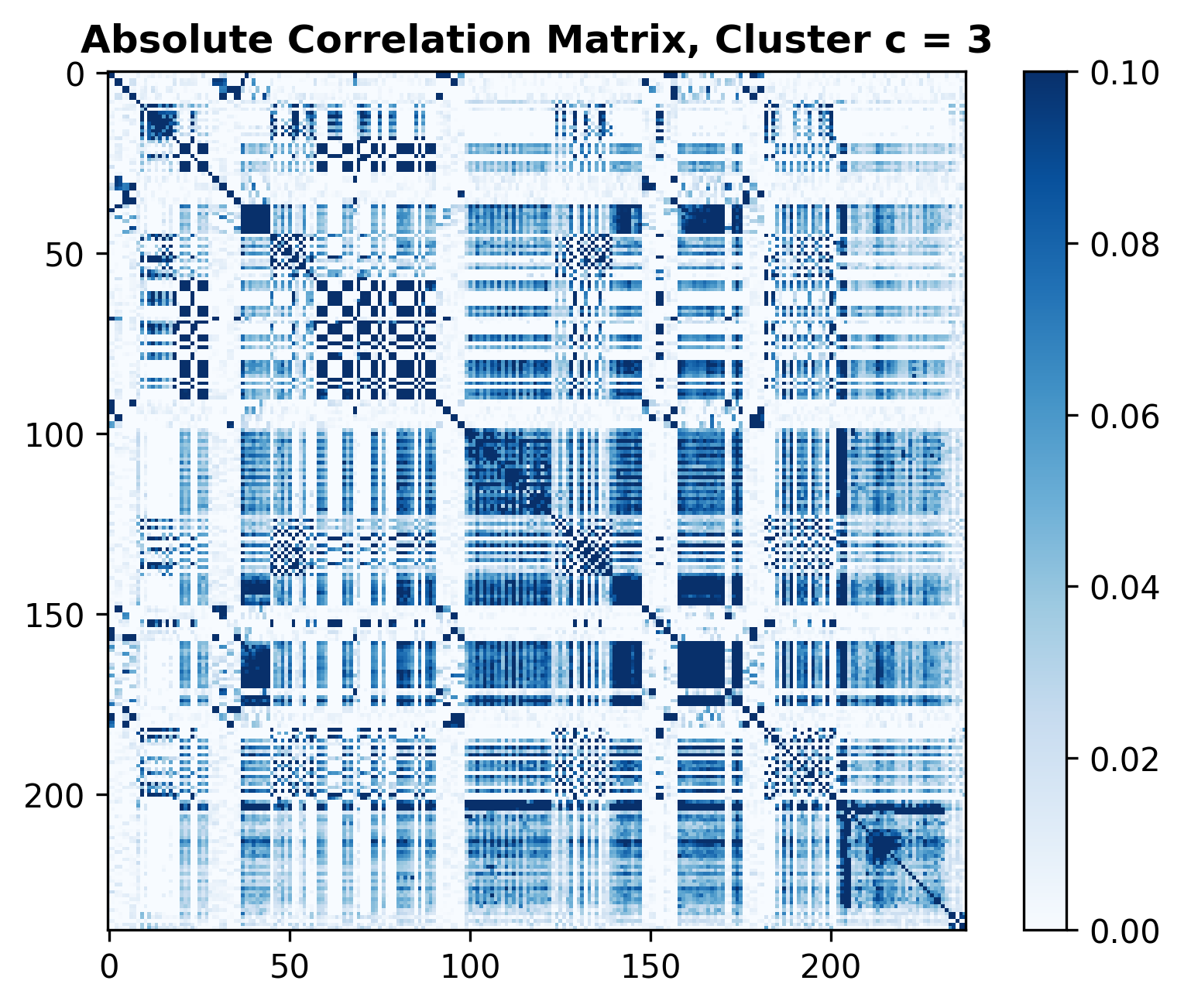

Supplement: Supplementary file 1 — Supplementary Material 1. [file 12874_2025_2487_MOESM1_ESM.zip › corr-matrix-clus3.png]

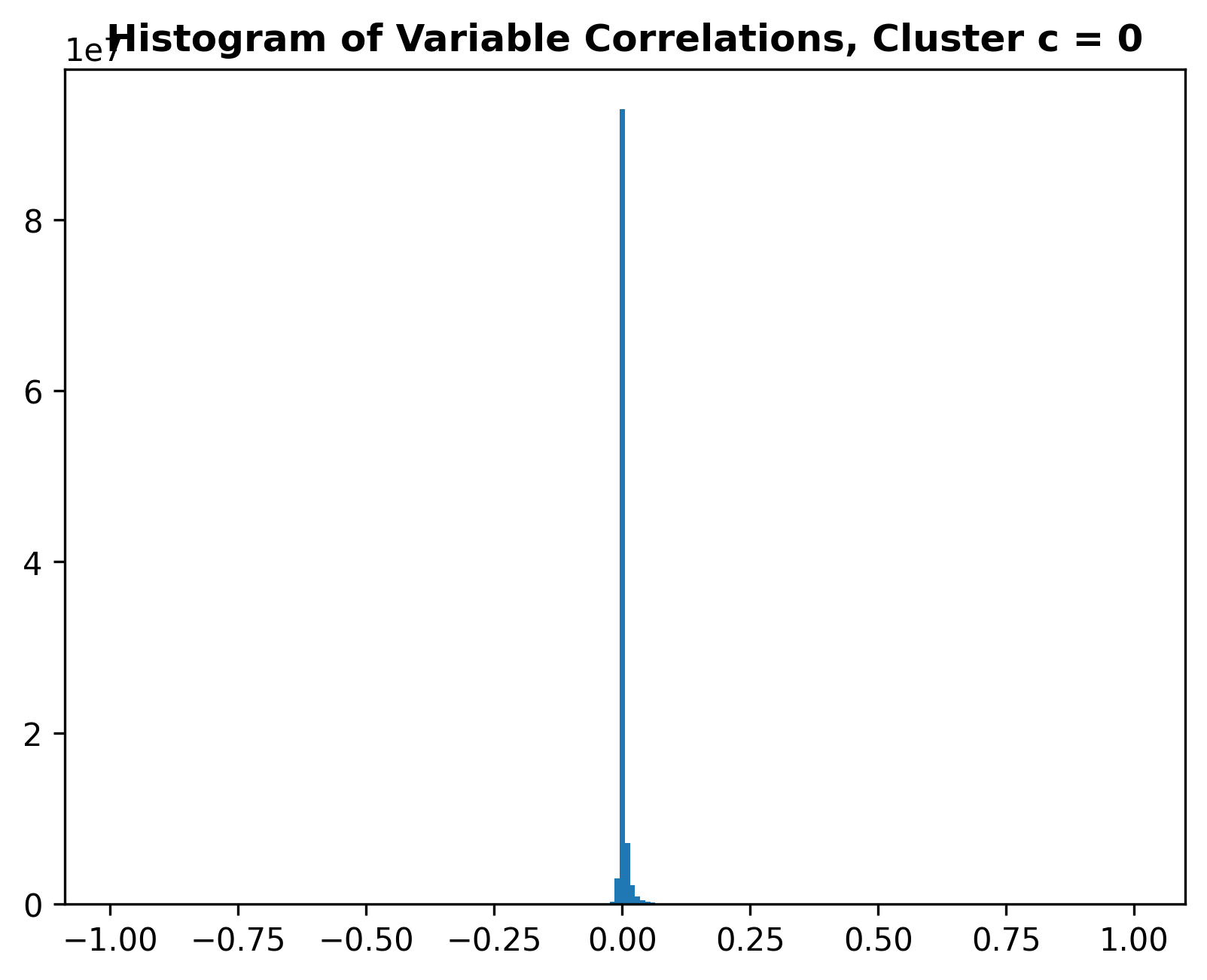

Supplement: Supplementary file 1 — Supplementary Material 1. [file 12874_2025_2487_MOESM1_ESM.zip › hist-corr-clus0.png]

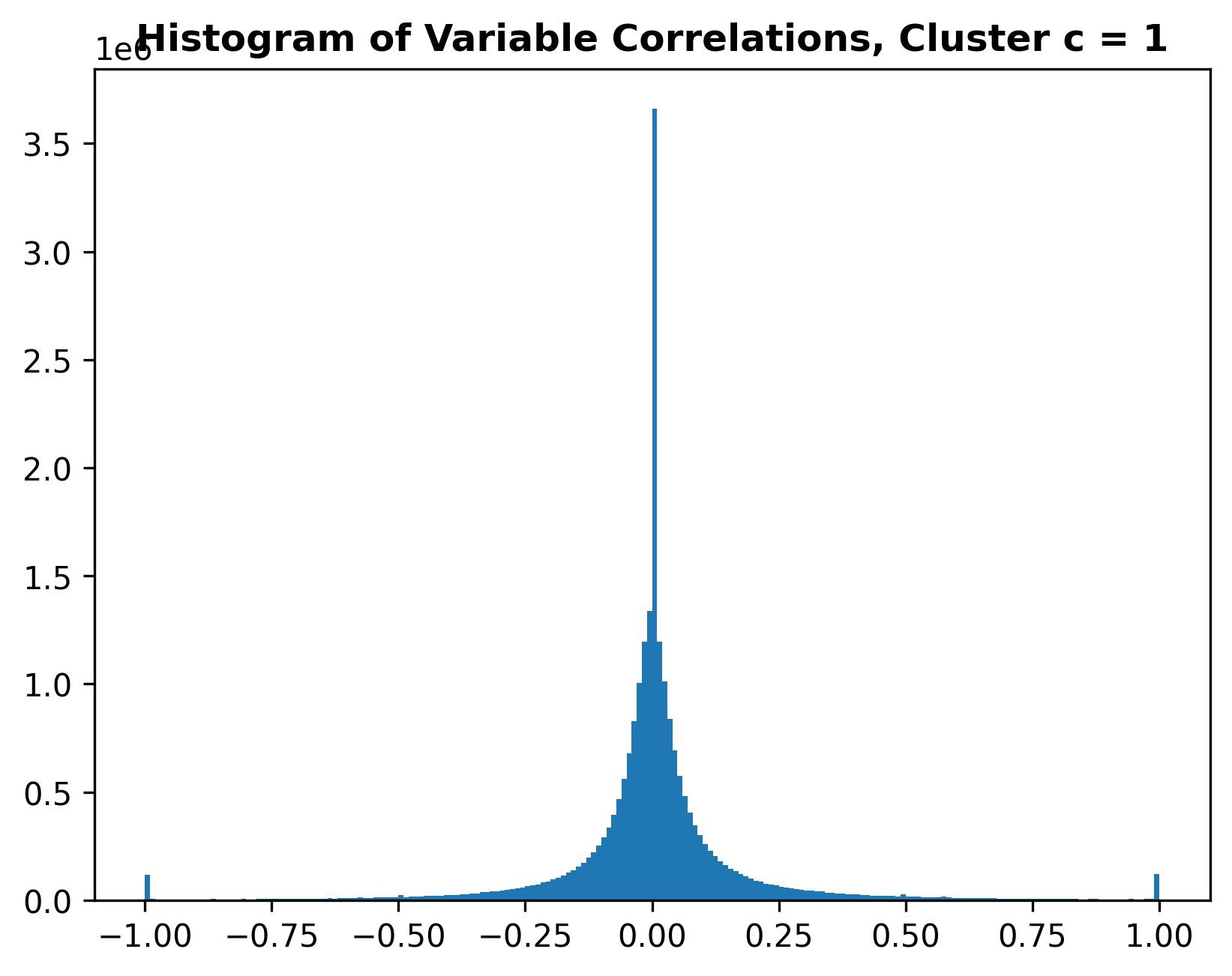

Supplement: Supplementary file 1 — Supplementary Material 1. [file 12874_2025_2487_MOESM1_ESM.zip › hist-corr-clus1.png]

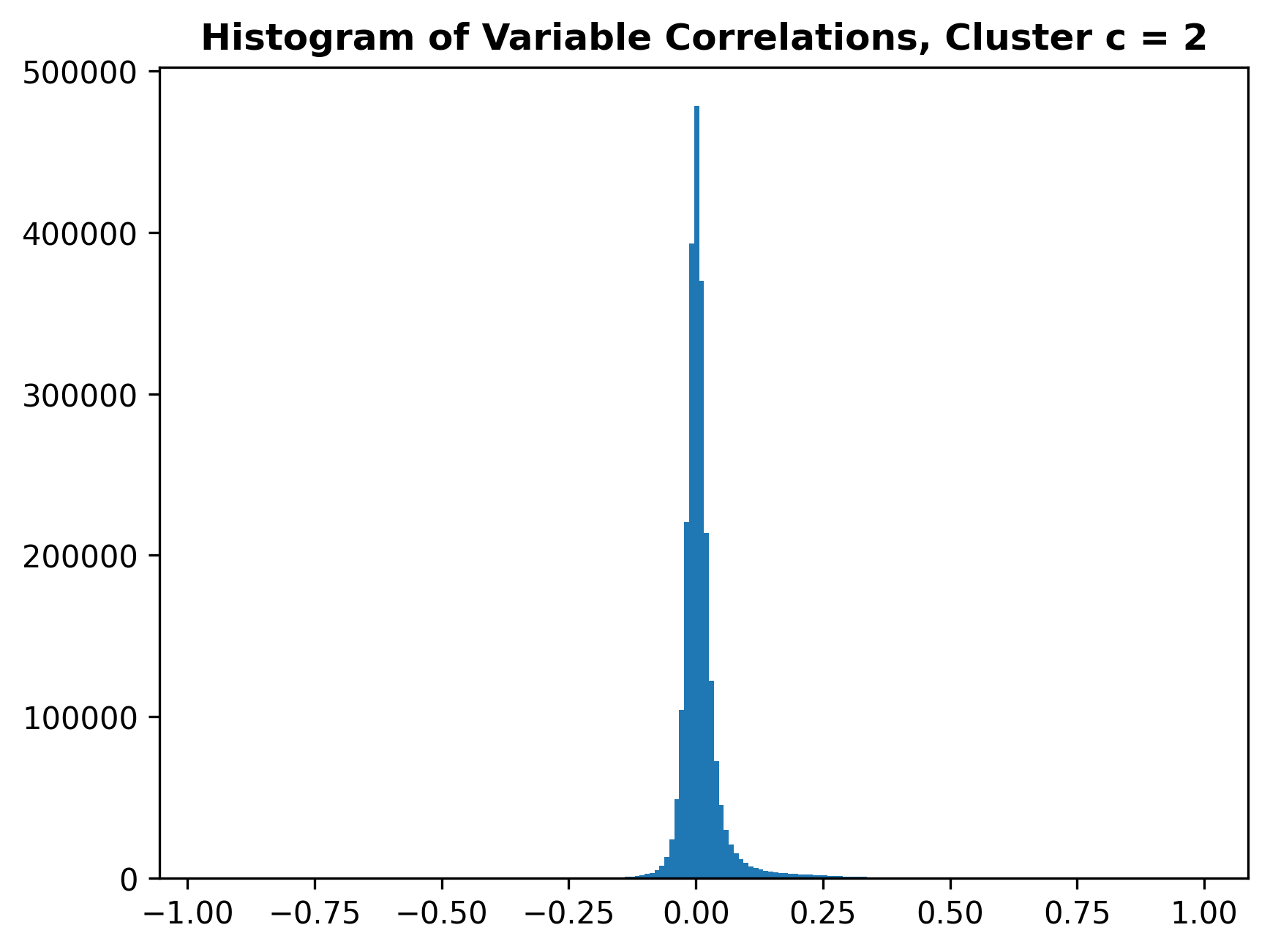

Supplement: Supplementary file 1 — Supplementary Material 1. [file 12874_2025_2487_MOESM1_ESM.zip › hist-corr-clus2.png]
